# Morphology of Graphene Aerogel as the Key Factor: Mechanical Properties Under Tension and Compression

**DOI:** 10.3390/gels11010003

**Published:** 2024-12-25

**Authors:** Elizaveta Rozhnova, Julia Baimova

**Affiliations:** 1 Physical-Technical Institute, Ufa University of Science and Technology, Z. Validi 32, Ufa 450076, Russia; rozhnova.elizavetaa@mail.ru; 2 Institute for Metals Superplasticity Problems of the Russian Academy of Sciences, Ufa 450001, Russia; 3 World-Class Research Center for Advanced Digital Technologies, Peter the Great St. Petersburg Polytechnic University, St. Petersburg 195251, Russia

**Keywords:** graphene honeycomb, molecular dynamics, strength, compression

## Abstract

Graphene aerogels with high surface areas, ultra-low densities, and thermal conductivities have been attracted a lot of attention in recent years. However, considerable difference in their deformation behavior and mechanical properties lead to their poor performance. The problem can be solved by preparing graphene aerogel of given morphology and by control the properties through the special structure of graphene cells. In the present work, molecular dynamics simulation is used to overview the mechanical properties of four different morphologies of graphene aerogel: honeycomb, cellular, lamellar and randomly distributed graphene flakes. All the structures are considered under uniaxial compression and tension with the detailed analysis of the deformation behavior. It is found that cellular structures have much better compressibility and elasticity. During both compression and tension, cellular structures can be transformed from one to another by controlling the compression/tensile direction. The highest strength and fracture strain are found for the lamellar GA under tension along the direction perpendicular to the alignment of the graphene walls. This reveals that the mechanical properties of graphene aerogels can be controlled by enhancing the structural morphology. The obtained results is the contribution which provide the insights into recent developments concerning the design of carbon-based structures and their application.

## 1. Introduction

Graphene aerogel (GA) is a three-dimensional carbon structure with the skeleton walls that can be approximately regarded as ultrathin graphene films (nanoribbons). GAs demonstrates high porosity, ultralight weight, and very unusual deformation behavior, which can be used for different applications ranging from pollutant adsorption to energy storage, catalyst support, supercapacitor, and thermal insulation, etc. [1,2,3,4,5,6]. For the first time, carbon aerogel was synthesized through the pyrolysis and carbonization of organic gel in 1990 [7]. Further, such aerogels have been obtained by self-assembly or gelation of the graphene oxide suspension via hydrothermal reduction, chemical reduction, or direct crosslinking of the graphene [8,9,10,11]. The other very effective approach is the 3D printing of GAs with the given morphology [12,13], chemical reduction [14], chemical cross-linking [15], to name a few. One of the promising directions for the application of GAs is the fabrication of composites with enhanced mechanical and thermal properties [16,17,18].

The properties of GAs are highly dependent on their particular morphology, for example, the quality of graphene and the density of compaction of the skeleton are crucial for heat conduction [17,19]. The residual oxygen-containing functional groups in GAs are also an important factor in determining the mechanical properties [20]. For different GAs, thermal conductivity varies in a wide range from very low 4.7 × $10^{-3}$–5.9 × $10^{-3}$ W/m · K due to the higher thermal contact resistance at the interfaces between adjacent graphene nanoribbons [21] to 28 × $10^{-3}$–39 × $10^{-3}$ W/m · K [22]. It is still a great challenge to improve the thermal conductivity of such 3D graphene network to achieve a high through-plane thermal conductivity of more than 10 W/m · K. Taking into account the high compressibility (strain > 50%) [23], recoverability [24,25] and unusual tensile behavior [26], the properties can be controlled by the structure modification. Thus, optimizing the structure (pore structure, lattice structure, and microstructure) of GAs is a promising way to improve the applicability of GAs [2].

The compressibility and elasticity of GAs are mainly determined by the intrinsic feature of the building blocks, and the structural design of the porous network [27]. GAs demonstrates high flexibility and superelasticity [28], but can be mechanically fragile under certain conditions, which leads to the collapse of the pores, changes of the specific surface area and consequently to the property degradation [6,29]. Understanding how to improve the mechanical characteristics is of crucial importance for the future application of GAs. Most of the research on GAs has focused on their synthesis and the experimental characterization of their superior properties; however, the study of the relationship between the promising mechanical properties and their unique structures is still very limited.

In [6,30], the review on the comparison of different morphologies of GAs and their mechanical properties is presented. Despite the benefits of understanding of the mechanical properties of GAs and the numerous published articles on the subject [6,23,27,28], a literature review reveals a limited number of comparative analyses on the role of the morphology of GAs. It turned out that existing reviews [30] cover a specific morphology, describing the syntheses, properties and the application, lacking in-depth discussions on the deformation mechanisms and mechanical properties in comparison. As was shown in [6,27], the graphene flake (GF) size, density of GA, surface functional groups, etc.; have a great influence on the mechanical performance of the GAs, especially on the compressibility and elasticity. These structures show excellent elasticity [23,31,32].

To provide a novel approach for in-depth investigations of the mechanical properties for different structures and to show the main differences, and find the affecting structural factors, molecular dynamics (MD) simulation can be effectively used [26,29,33,34,35]. In [29], it was found by MD that up to high strain, no stress increase is observed due to the structural transformation, and significant stress increase occurs only at high strain due to the stress concentration over graphene walls. In [35], the mechanical properties of nanoporous graphene network have been investigated experimentally and additionally described by MD. It is revealed that high plasticity can be transformed into high elasticity by high temperature heat treatment. For Schwarz-surface-like GA it has been explained by MD how to improve the mechanical properties of GA by enhancing the structural continuity [34]. In [33] MD simulations were used to study the 3D GA system and demonstrate its superior mechanical properties compared to most polymeric cellular materials. The work [26] focused on the effect of aspect ratio on the fracture strength and toughness of GA.

This work is the continuation of work [29] on the mechanical properties of honeycomb and re-entrant honeycomb GAs, where the differences in the deformation behavior of the simplest honeycombs were revealed. As was previously found, structure of the GA define its mechanical behavior. It was shown that honeycomb and re-entrant honeycomb can sustain much higher deformation than the other morphology; however, the mechanical properties have been characterized only for three simple morphologies under tension. The results revealed that it is important to expand this study to other common morphologies with a full description of the deformation behavior, including compression and tension with the characterization of the microstructure. In the present work, four different structures are compared under uniaxial tension and compression. The detailed analysis of the microstructure and the search for the main structural characteristics of such GAs are performed by MD simulation.

## 2. Results and Discussion

### 2.1. Hydrostatic Compression

**Figure 1:**
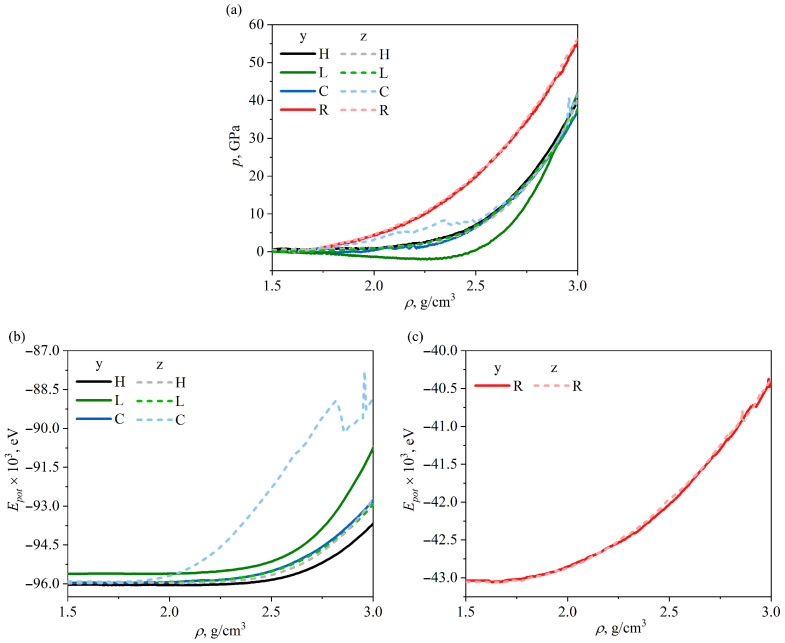
(**a**) Pressure-density curves during hydrostatic compression along *y*-axis (solid lines) and *z*-axis (dashed lines) for all structures under consideration. (**b**,**c**) Potential energy as the function of density. All the results are presented for H (honeycomb), L (lamellar), C (cellular), and R (random) GAs.

Figure 1a presents the pressure-density curves obtained during the compression of the GA along the *y*-axis (solid lines) and the *z*-axis (dashed lines). It is observed that the pressure is zero when ρ < 1.75 g/${\mathrm{cm}}^{-3}$, for all the considered GAs. This happens due to the collapse of the cells. For such GAs, it is better to consider the density of the structure rather than the strain, because due to the pores achieved strain can be very high. The curves for H, L and C can be divided into four parts: zero stress, a linear elastic, yielding and densification parts, which are similar observations to those of other works on the compression of silica and carbon aerogels [26,36,37]. In all cases, the rigidity of the GA increases with its density, due to the formation of new van der Waals bonds (for H, L and C) and covalent bonds between neighboring GFs (for R). This behavior is explained by the special mechanical response of the graphene network to the compression [37]. For compression to density ρ > 3.0 g/${\mathrm{cm}}^{-3}$, amorphization of the GA occurred.

As can be seen, there is a slight anisotropy in the compression behavior of L and C GAs along the *y* and *z* axes. Negative pressure during compression along *y*-axis for lamellar GA appeared due to the oscillation of the cell walls (see Figure 1a). Much faster stress increase for cellular GA during compression along *z*-axis is explained by (see Figure 1). Random and honeycomb GAs are isotropic.

From Figure 1b,c, it can be seen that the potential energy as a function of density can also characterize the structural changes. For three similar GAs (lamellar, honeycomb and cellular), the potential energy is almost unchanged up to ρ = 2.2 g/${\mathrm{cm}}^{3}$. Only for cellular GA for compression along *z*-axis, a considerable increase of the potential energy occurs at ρ = 2.0 g/${\mathrm{cm}}^{3}$, which is explained by the strong folding of the cell walls. The equilibrium density of the structure can also be determined from the potential energy curves: a rapid increase in energy corresponds to the formation of the rigid structure. From Figure 1c it can be seen, that the initial density of random GA was equilibrium and further compression leads to rapid folding and crumpling of the GFs with increasing of interaction forces.

**Figure 2:**
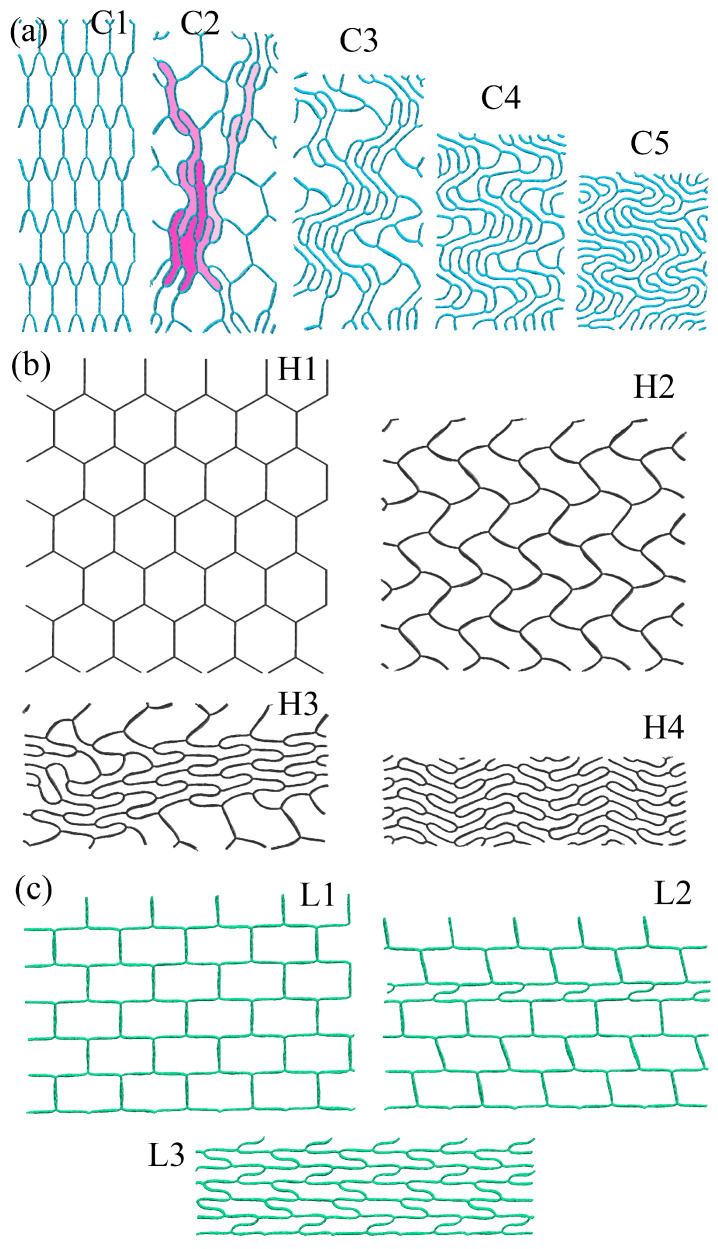
Snapshots of the GA during compression along *y*-axis: (**a**) cellular (C); (**b**) honeycomb (H); and (**c**) lamellar (L). The densities ρ of the structures are as follows, in g/${\mathrm{cm}}^{3}$: (**a**) C1—0; C2—1.2; C3—1.5; C4—2.2; C5—3.0. (**b**) H1—0; H2—1.2; H3—1.5; H4—2.5. (**c**) L1—0; L2—1.7; L3—2.7.

Let us first consider the compression along the *y*-axis. In this case, the pressure-density curves for H and C are coincide, the compression goes through the same way until ρ = 3.0 g/${\mathrm{cm}}^{3}$. To better analyze this process, the structural transformations during compression are presented in Figure 2.

Figure 2 presents the snapshots of the GA during compression at different strain levels. As can be seen, for honeycomb and cellular GA, the pressure was equal to zero up to ρ = 2.2 g/${\mathrm{cm}}^{3}$ which is related to the continuous collapse of the hexagons (for H) and the opening of the hexagons (for C). The initial compression causes the mild flexing of the structure, followed by a series of collapses of cells, leading to an eventual flattening for cellular and honeycomb GAs. Folding and crumpling occurred for all the considered structures. During compression mainly bond angles are changing to the values lower or higher than the initial $120^{\circ }$ equilibrium structure. For cellular GA, some of the hexagons are opened, while the others are collapsed: in Figure 2 C2, the first collapsed hexagons are shown in light pink; the second—in pink; and the last—in dark pink. During further compression, more hexagons collapsed and rotated (at ρ = 1.5 g/${\mathrm{cm}}^{3}$, C3). The mechanism of cell collapse is very analogous to the collapse of carbon nanotubes: two main stable structural states can be possessed—open and collapsed nanotubes [38]. The strain increase starts at ρ = 2.2 g/${\mathrm{cm}}^{3}$ (C4). The rapid strain increase is explained by the complete collapse of the hexagons, and at ρ = 3.0 g/${\mathrm{cm}}^{3}$ all graphene walls are 3.4 Å far from each other. Further strain increase leads to amorphization.

The same is found for honeycomb GA: continuous collapse of hexagons with their rotation, without any stress increase. The structure shown in H3 corresponds to ρ = 1.5 g/${\mathrm{cm}}^{3}$, while H4 corresponds to ρ = 2.5 g/${\mathrm{cm}}^{3}$: when all the hexagons are collapsed, a rapid stress increase occurred. This would be very similar to the compression along the *z*-axis: hexagons continuously collapsed and rotated. So, no snapshots of the structure are presented.

For lamellar GA, the stress is even below zero, because during compression rectangles are collapsed line by line until ρ = 2.7 g/${\mathrm{cm}}^{3}$ (L3). Such collapse allowed the structure to relax. Finally, all the honeycomb structures reached almost the same structural state.

There is a slight difference in the deformation behavior of cellular GA during compression along the *z*-axis. Until ρ = 1.7 g/${\mathrm{cm}}^{3}$, half of the hexagons are collapsed, and further stress increase is associated with these strained walls. Up to ρ = 2.1 g/${\mathrm{cm}}^{3}$ all the hexagons have collapsed and the stress has increased. The stress increases due to the formation of rigid folds, but the increase is slow because of the mutual sliding of the hexagons. At ρ = 2.5 g/${\mathrm{cm}}^{3}$, the folds have become even more rigid, there is no empty space for sliding and the stress increases rapidly.

In the GA with random distribution of GFs, the slow stress increase can be explained by the pore removal during the first compression steps, mutual alignment of the flakes in the structure and formation of folds and wrinkles. The structure is not shown because the visual analysis cannot give clear explanations. As was found, the number of covalent bonds across the edges of neighboring GFs increases during compression. Faster strain increase after ρ = 2.2 g/${\mathrm{cm}}^{3}$ is explained by the formation of a large number of van der Waals bonds between the flakes and the increase of the flakes rigidity due to their folding.

### 2.2. Uniaxial Tension

**Figure 3:**
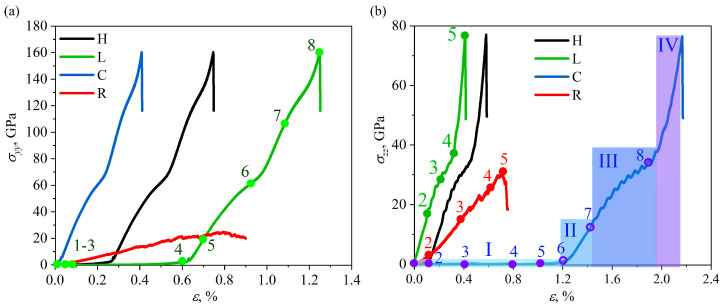
Stress-strain curves under uniaxial tension along *y*-axis (**a**) and *z*-axis (**b**) for all structures under consideration. The main points on the stress-strain curves are labeled by dots and numbers 1–8. Structural states at these points are further analyzed. Stress-strain curve for cellular GA is divided into four regions I–IV to characterize the different deformation mechanisms. All the results are presented for H (honeycomb), L (lamellar), C (cellular), and R (random) GAs..

Figure 3 presents the stress-strain curves for GAs under tension along the *y* and *z* axes. Ultimate tensile strength of H, L and C GAs depends significantly on the tensile direction, while R GA is again isothropic. Ultimate tensile strength of H, L and C aerogels is two times higher for tension along *y*-axis, than along *z*-axis. Several important characteristics can be revealed: ultimate tensile stress $\sigma_{UTS}$ and fracture strain $\varepsilon_{F}$, at which the fracture occurred; pre-critical stress $\sigma^{*}$, after which rapid stress increase took place. Young’s modulus can be defined from the linear region. The obtained values of Young’s modulus, tensile strain and strength are presented in Table 1.

As can be seen, the curves for H and L under tension along the *y*-axis and for H and C under tension along the *z*-axis can be divided into four regions: zero stress; linear elastic regime; inelastic deformation and pre-critical stress increase. These deformation stages are represented by blue regions in the example of cellular GA under tension along *z*-axis. For cellular GA under tension along *y*-axis and lamellar GA under tension along *z*-axis, there is no region with zero stress level. All these differences and similarities are explained by the intrinsic structure of GA, which will be discussed further.

**Figure 4:**
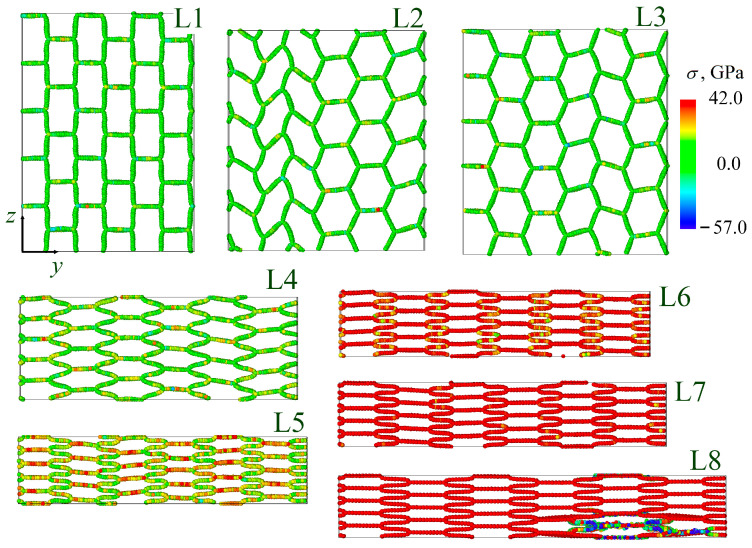
Stress per atom $\sigma_{yy}$ during tension along *y*-axis for lamellar (L) GA. Number corresponds to the numbers on the stress-strain curves in Figure 3.

**Figure 5:**
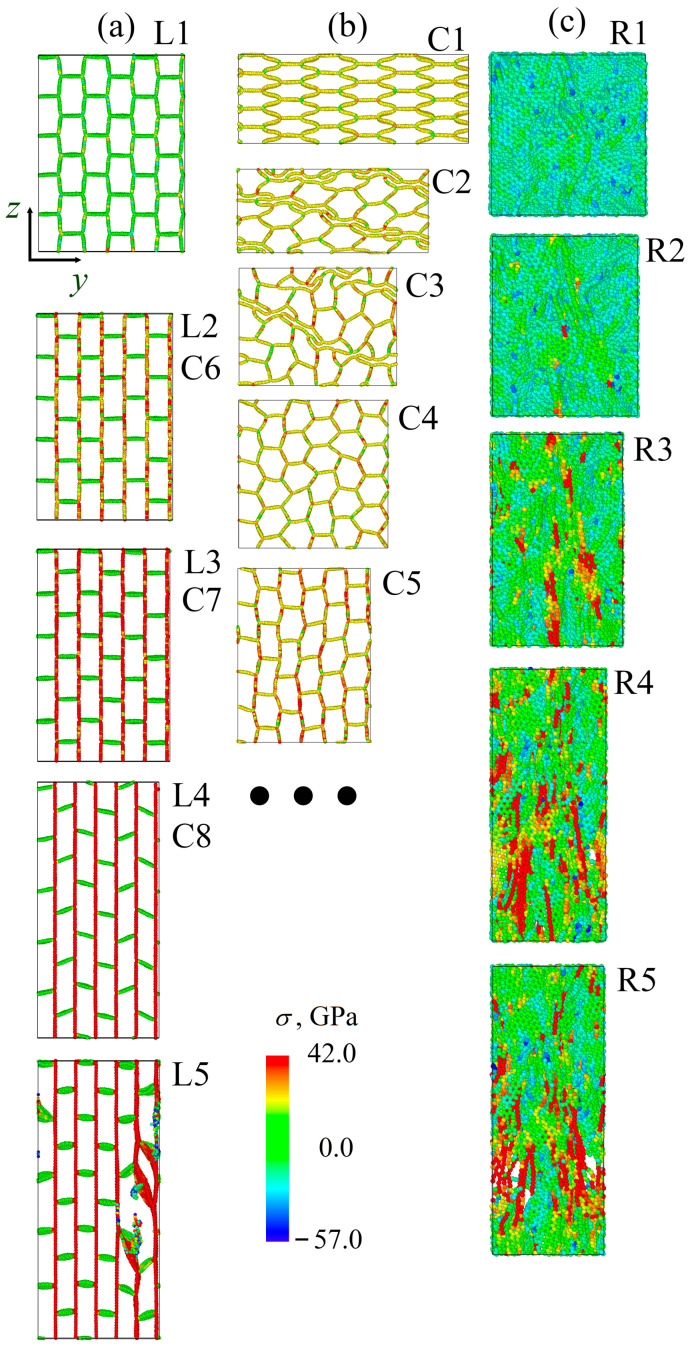
Stress per atom $\sigma_{zz}$ during tension along *z*-axis. Here, L—lamellar GA, R—random GA, C—cellular GA. Number corresponds to the numbers on the stress-strain curves in Figure 3.

Figure 4 and Figure 5 present the stress per atom $\sigma_{yy}$ ($\sigma_{zz}$) during tension along *y*-axis (*z*-axis) of all the GAs under investigation. For deformation along *y*-axis, all the structural changes can be explained based on the snapshots for lamellar GA (corresponding strain is marked by points in the stress-strain curve). At first, rectangular cells start to transform to the hexagonal. Oscillation of the hexagons took place up to ε = 0.5: some are rotated (L2), some are opened (L3), with continuous changing of their shape. Finally, all the cells transform to the hexagons (L3) and start to collapse during further tension (L4). For all the considered structures, folding and buckling of the graphene occurred. From point L3 we can also describe the deformation behavior of the honeycomb GA (black curve in Figure 3a). From point L4 we can describe also the deformation behavior of cellular GA (blue curve in Figure 3a). At ε = 0.6, the hexagons cannot be collapsed any further and the graphene nanoribbons aligned with the tensile direction are stressed. Further, deformation is mainly defined by the stretching of the walls of GA cells parallel to the tensile direction. The initial length of the wall is 12 Å and unchanged up to L5. It is increased to 16 Å before the fracture occurred. The analysis of the interatomic bonds inside the basal plane of graphene walls showed that for walls along the *x*-axis, the lattice parameters remained almost unchanged until L5. The bonds almost aligned with the loading direction are mostly strained. These bonds (and others of the same orientation) are continuously changing from 1.41 Å to 1.7 Å. Point L6 is the pre-critical stress, when all the graphene walls are considerably loaded. Fracture occurred on one of the graphene walls aligned with the tensile direction.

Let us discuss the deformation behavior along the *z*-axis for lamellar, cellular and random structures (see Figure 5). Again, the deformation behavior of H, L and C GAs can be described by the same snapshots. The highest elasticity is found for cellular GA because during the first stages up to ε = 1.2, different structural changes took place: opening and rotation of the cells, oscillations of the graphene walls, transformation to hexagonal GA (point C4) and further transformation to lamellar GA (C6, L2). The most stressed walls are aligned with the tensile direction, while the normal graphene walls (colored green in Figure 5) first oscillate, and then slightly, allowing further deformation.

For random GA, the deformation behavior is similar along *y* and *z* axes and also in good agreement with the previously known [26,33]; however, in the present work GA with the high density is considered, which can considerably affect the strength. This is the GA with the lowest strength and fracture strain, because, compared to cellular graphene, not all the GFs are bonded by covalent bonds. GFs are connected to each other at the edges, but mostly, van der Waals interaction prevails. It can be seen, that after the linear region is finished, some GFs became stressed (shown by red in R3). Further, GFs can transform to the carbon chains (red lines in R4). Formation of such carbon chains is a well-known deformation mechanism for graphene and allows to increase fracture strain of GA. At point R5, fracture of most of the carbon chains occurred with the failure of the structure.

**Figure 6:**
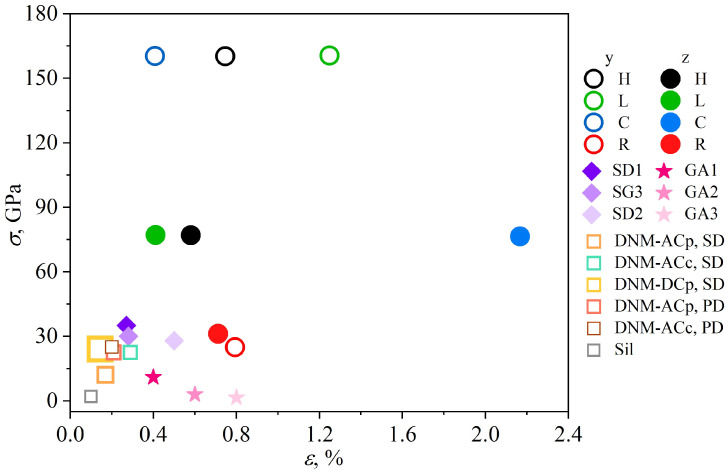
Ultimate strength and fracture strain of GA configurations in comparison with other results (circles). Tensile strain and strength for SD1, SG3, SD2 are taken from [34], for GA1-GA3 from [26], for Sil from [36], for DNM from [39]. The obtained in the present work results are presented for H (honeycomb), L (lamellar), C (cellular), and R (random) GAs.

Figure 6 presents the overview of the fracture strain and strength of different GAs for comparison. Five diamond nanomeshes [39], silica aerogel [36], GAs with different densities from [26], continuous curved GAs [34] are compared by their ultimate tensile strength and strain. GAs studied in [26] are even weaker (pink stars) than those studied in the present work (red circles), because of the two times lower density of GA. In contrast, very similar discontinuous GAs failed by the fracture strain, because they have a more rigid structure with pre-defined interconnections between GFs. It reveals, that random distribution of GFs in random GA results in better strength and fracture strain. Moreover, the higher the density of such GA, the higher the ultimate tensile strength under tension, but the lower the compressibility.

Various GAs, composed of diamond nanothreads (square signs) are also much weaker than pure graphene structures. Even the changes in the structural morphology cannot results in the strain increase. At the same time, honeycomb, cellular and lamellar GAs have a lot in common: high compressibility, high elasticity, ability to transform from the one structure to another. Ultimate tensile strength and fracture strain, as well as the Young’s modulus are highly dependent on the tensile direction. The highest strength and fracture strain demonstrates the lamellar GA under tension along the direction perpendicular to the alignment of the graphene walls: this allows the transformation from lamellar to honeycomb and further to cellular structure. This also provides an opportunity to tune the structural morphology and properties of the GAs.

Some comparison of the obtained results on the mechanical properties of GA under compression with the literature are presented in Table 2.

Besides the morphology of GA, many different factors can also affect their mechanical properties which should be analyzed further. For example, oxygen-containing functional groups can considerably affect the mechanical properties due to π−π stacking interactions between graphene sheets [20]. As was shown in [40], for lamellar structures, especially with the square cells, the length of the cell walls plays a crucial role both in compression and tension: the longer the graphene walls, the lower the ultimate strength and strain: GA with short bridges can absorb more strain than specimens with longer bridges. In the frames of the present work, only pure GAs are considered for simplicity.

## 3. Conclusions

In the present work, we analyzed a group of GAs under tension and compression using molecular dynamics simulation. A full-atomic GA models with different densities and structures is developed to thoroughly investigate the mechanical responses of GAs of different morphologies. The porous morphology and density are key factors determining the mechanical response of GA.

Upon compression, the GAs remained elastic up to the densities of about 1.7–2.0 g/${\mathrm{cm}}^{3}$ in the *y* and *z* directions. The honeycomb and random GAs demonstrates the same deformation behavior along two compression directions, while the lamellar and cellular structures show slightly anisotropic deformation upon compression. In tension along *y*-axis, elastic strains are observed up to ε = 0.7 for lamellar, up to ε = 0.35 for honeycomb, up to ε = 0.1 for cellular structure. Maximum ultimate strengths of 160 GPa in tension in the *y* direction for honeycomb, cellular and lamellar GAs and 25 GPa for random GA are observed, with each loading case having its own characteristic stress-strain curve. In tension along the *z*-axis, elastic strains are observed up to ε = 0.1 for lamellar, up to ε = 0.3 for honeycomb, up to ε = 1.3 for cellular and up to 0.3 for random. Maximum ultimate tensile strengths of 77 GPa in tension in the *z* direction for honeycomb, cellular and lamellar GAs and 31 GPa for random GA are observed. All the considered structures exhibit highly anisothropic behavior in tension along the *y* and *z* axes.

GAs demonstrated interesting, highly nonlinear deformation behavior under tension and compression in both the *y* and the *z* directions. In the early stages of deformation, the graphene walls of the GA cells can be easily folded and oscillate in the direction normal to the tension/compression direction. At high deformation levels, the stress is concentrated near the junctions of the GA and along the walls oriented along the tensile direction. The deformation is defined by the opening/collapsing of the cells during deformation and depends on the particular morphology of the GA. The important point is that GA can transforms during tension and compression from one morphology to another, which allows the possibility of property comtrol by changing the special morphology. Mostly, the structural transformations are reversible and can be achieved by both tension and compression. Analysis of the microstructure showed that the graphene walls aligned with the tensile direction are the most stressed.

Understanding of the dependence of different mechanical properties on the morphologies of GAs is important for the future applications of such structures. Further studies can be devoted to the analysis of how to improve the compressive properties of GAs by structural modification, for example by adding reinforcing elements.

## 4. Materials and Method

**Figure 7:**
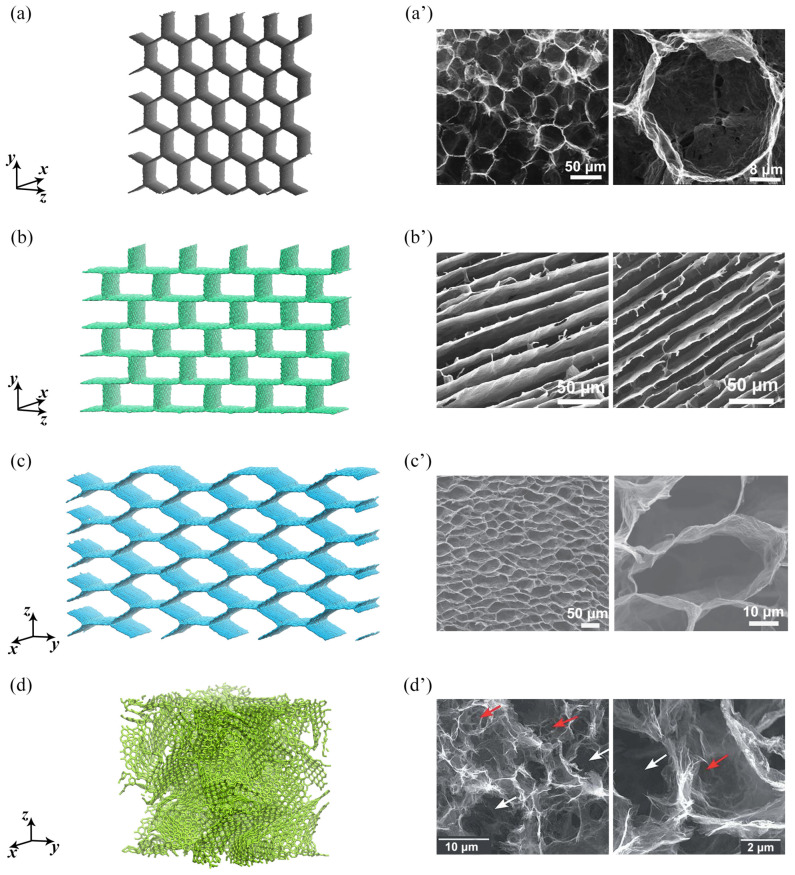
(**a**,**a’**) H—honeycomb GA; (**b**,**b’**) L—lamellar GA; (**c**,**c’**) C—cellular GA; and (**d**,**d’**) R—GA with randomly oriented GFs. Here, (**a**–**d**) are the model representation of (**a**’–**d**’) experimentally synthesized GAs. (**a’**–**d’**) are adopted with the permission from [17,41,42,43], respectively.

Figure 7 presents the GAs under consideration: Figure 7a,a’ H—honeycomb; Figure 7b,b’ L—lamellar; Figure 7c,c’ C—cellular; and Figure 7d,d’ R—GA with randomly oriented GFs. Here, Figure 7a–d are the model representations of Figure 7a’–d’ experimentally synthesized GAs, each adopted with the permission from [17,41,42,43]. Interestingly, very similar porous carbon honeycomb structures of different morphology can be derived from hemp [44]. Structures (a–d) are the initial GAs, that would be further considered.

As can be seen in Figure 7, three of the considered GAs have ordered pores: hexagonal (a); rectangular (b); and collapsed hexagonal (c). These structures can be characterized by the length of the cell wall: 12.5 Å. The last GA (d) have pores random by size and distribution.

To obtain any of the studied GA, graphene nanoribbons are combined by the home-made program: one graphene nanoribbon is aligned along xy-plane, then four adjacent graphene nanoribbons are rotated at different (depending on the morphology) angle α and connected with the first one. At the triple junctions, $sp^{3}$-hybridization is achieved, structure is carefully checked for proper interconnection between carbon atoms in the junction. All the initial structures are generated by the homemade program, which allows to generate the graphene cellular structure with different cell morphology, different size, etc.

GA with random structure is made of 125 GFs of the same size. The GFs are randomly rotated and combined to form the whole structure. First, hydrostatic compression at 1000 K is applied to obtain the GA with covalent bonds between GFs. At the beginning of the simulation, the GFs are randomly oriented and distributed.

The size of the structures and other main parameters are presented in Table 3. Specific surface area (SSA) which is a total surface area *S* of a material per unit mass *m* can be calculated as SSA = S/m. The density of the experimentally obtained GAs is also presented in Table 3 as $\rho^{*}$ [17,41,42,43].

First, the obtained GAs are equilibrated with the NPT ensemble at 0.01 K. Then, uniaxial tensile/compressive stress at 300 K is applied to the composites along the *y*- and *z*-axes, and the corresponding stress components are calculated. In this work, all the mechanical tests are performed with a quasi-static loading. The strain rate is $\dot{\varepsilon }$ = 0.001 ${\mathrm{ps}}^{-1}$.

The interaction between carbon atoms is described using the well-known AIREBO potential [45]. Previously, this potential has been successfully used to study the formation of pure carbon nanoparticles [46], the impact-induced penetration of GA [47], the deformation behavior of GA [26,29], to name a few.

AIREBO potential can be written as

(1) $$U_{C-C}=\frac{1}{2}\sum_{i}\sum_{i\neq j}\left[U_{ij}^{REBO}+U_{ij}^{LJ}+\sum_{k\neq i,j}\sum_{l\neq i,j,k}U_{kijl}^{TORSION}\right],$$

where $U_{ij}^{REBO}$ is the hydrocarbon REBO potential, $U_{ij}^{LJ}$ term adds longer-ranged interactions using a form similar to the standard Lennard-Jones potential, and $U_{kijl}^{TORSION}$ describes various dihedral angle preferences in hydrocarbon configurations.

All the simulations are conducted using MD simulations in the Large-scale Atomic/ Molecular Massively Parallel Simulator (LAMMPS) simulation package [48,49,50]. The equations of motion for the atoms are integrated using the fourth-order Verlet method with a time step of 0.2 fs. Periodic boundary conditions are applied along the *x*-, *y*-, and *z*-axes. The Nose-Hoover thermostat is used to control the system temperature when simulating the relaxation and tension. For the structure visualisation, the molecular graphics programs Visual Molecular Dynamics (VMD) and Open Visualization Tool (OVITO) are used [51,52].

## Figures and Tables

**Table 1:** Young’s modulus *E*, ultimate tensile strength strength $\sigma_{UTS}$, fracture strain $\varepsilon_{F}$ and pre-critical stress $\sigma^{*}$ for GAs under tension.

Tensile Direction	Structure	$\sigma_{\mathrm{UTS}}$, GPa	$\sigma^{*}$, GPa	$\varepsilon_{F}$	*E*, GPa
*y*-axis	H	160.25	67.38	0.74	308.19
	L	160.62	67.06	1.24	243.21
	C	160.40	67.28	0.40	361.15
	R	24.92	–	0.79	32.27
*z*-axis	H	77.04	34.96	0.58	131.74
	L	77.11	35.63	0.41	145.62
	C	76.51	34.05	2.16	64.14
	R	31.15	–	0.71	43.63

**Table 2:** GA under compression: density and compressive stress.

Structure	Density, g/${\mathrm{cm}}^{3}$	Compressive Stress, GPa	Ref.
R	1.0	5	[35]
R	0.7	4	[35]
SP1	0.838	4	[34]
SG3	0.803	8	[34]
SD2	0.792	13	[34]
R	2.0	6	our work
C	2.0	2	our work
L	2.0	1	our work
H	2.0	1.5	our work

**Table 3:** Structural parameters for the GA: $L_{x}\times L_{y}\times L_{z}$—size of the simulation cell, ρ—density of the GA, *N*—number of atoms, SSA—specific surface area and the density of the experimentally obtained GAs $\rho^{*}$ from [17,41,42,43].

Structure	$L_{x}\times L_{y}\times L_{z}$, Å	ρ, g/${\mathrm{cm}}^{3}$	*N*	SSA, $m^{2}$/g	$\rho^{*}$, mg/${\mathrm{cm}}^{3}$
H	62.5 × 112.1 × 107.5	0.6	26,100	1440	2.8
L	62.5 × 82.9 × 126.1	0.7	26,100	1440	16.6–4.0
C	62.5 × 140.2 × 51.7	1.1	26,100	1300	5.1
R	53.2 × 53.2 × 55.1	1.6	12,288	1083	-

## Data Availability

Data can be available on request due to the privacy restrictions.

## Abbreviations

The following abbreviations are used in this manuscript:

GA	Graphene Aerogel
MD	Molecular Dynamics
GF	Graphene Flake
HC	Hydrostatic Compression
SSA	Specific Surface Area

## References

[1] Mao J., Iocozzia J., Huang J., Meng K., Lai Y., Lin Z. (2018). Graphene aerogels for efficient energy storage and conversion. Energy Environ. Sci..

[2] Cao X., Zhang J., Chen S., Varley R.J., Pan K. (2020). 1D/2D Nanomaterials Synergistic, Compressible, and Response Rapidly 3D Graphene Aerogel for Piezoresistive Sensor. Adv. Funct. Mater..

[3] Zhang F., Liu J., Hu L., Guo C. (2024). Recent Progress of Three-Dimensional Graphene-Based Composites for Photocatalysis. Gels.

[4] Zhu Q., Ong P.J., Goh S.H.A., Yeo R.J., Wang S., Liu Z., Loh X.J. (2024). Recent advances in graphene-based phase change composites for thermal energy storage and management. Nano Mater. Sci..

[5] Sultanov F., Tatykayev B., Bakenov Z., Mentbayeva A. (2024). The role of graphene aerogels in rechargeable batteries. Adv. Colloid Interface Sci..

[6] Xia Y., Gao C., Gao W. (2022). A review on elastic graphene aerogels: Design, preparation, and applications. J. Polym. Sci..

[7] Pekala R., Alviso C., Kong F., Hulsey S. (1992). Aerogels derived from multifunctional organic monomers. J. Non-Cryst. Solids.

[8] Bi H., Yin K., Xie X., Zhou Y., Wan N., Xu F., Banhart F., Sun L., Ruoff R.S. (2012). Low Temperature Casting of Graphene with High Compressive Strength. Adv. Mater..

[9] Sun H., Xu Z., Gao C. (2013). Multifunctional, Ultra-Flyweight, Synergistically Assembled Carbon Aerogels. Adv. Mater..

[10] Worsley M.A., Charnvanichborikarn S., Montalvo E., Shin S.J., Tylski E.D., Lewicki J.P., Nelson A.J., Satcher J.H., Biener J., Baumann T.F. (2014). Toward Macroscale, Isotropic Carbons with Graphene-Sheet-Like Electrical and Mechanical Properties. Adv. Funct. Mater..

[11] Worsley M.A., Olson T.Y., Lee J.R.I., Willey T.M., Nielsen M.H., Roberts S.K., Pauzauskie P.J., Biener J., Satcher J.H., Baumann T.F. (2011). High Surface Area, sp2-Cross-Linked Three-Dimensional Graphene Monoliths. J. Phys. Chem. Lett..

[12] Zhu C., Han T.Y.J., Duoss E.B., Golobic A.M., Kuntz J.D., Spadaccini C.M., Worsley M.A. (2015). Highly compressible 3D periodic graphene aerogel microlattices. Nat. Commun..

[13] Wang J., Shi Z., Gong J., Zhou X., Li J., Lyu Z. (2024). 3D printing of graphene-based aerogels and their applications. FlatChem.

[14] Tong Y., He M., Zhou Y., Nie S., Zhong X., Fan L., Huang T., Liao Q., Wang Y. (2018). Three-Dimensional Hierarchical Architecture of the TiO_2_/Ti_3_C_2_T_x_/RGO Ternary Composite Aerogel for Enhanced Electromagnetic Wave Absorption. ACS Sustain. Chem. Eng..

[15] Shen J., Zhang P., Song L., Li J., Ji B., Li J., Chen L. (2019). Polyethylene glycol supported by phosphorylated polyvinyl alcohol/graphene aerogel as a high thermal stability phase change material. Compos. Part B Eng..

[16] Yang J., Qi G.Q., Bao R.Y., Yi K., Li M., Peng L., Cai Z., Yang M.B., Wei D., Yang W. (2018). Hybridizing graphene aerogel into three-dimensional graphene foam for high-performance composite phase change materials. Energy Storage Mater..

[17] Liu P., Li X., Min P., Chang X., Shu C., Ding Y., Yu Z.Z. (2020). 3D Lamellar-Structured Graphene Aerogels for Thermal Interface Composites with High Through-Plane Thermal Conductivity and Fracture Toughness. Nano-Micro Lett..

[18] Wu Y., An C., Guo Y., Zong Y., Jiang N., Zheng Q., Yu Z.Z. (2024). Highly Aligned Graphene Aerogels for Multifunctional Composites. Nano-Micro Lett..

[19] Gibson L.J., Ashby M.F. (2001). Cellular Solids.

[20] Minakshi M., Samayamanthry A., Whale J., Aughterson R., Shinde P.A., Ariga K., Kumar Shrestha L. (2024). Phosphorous–Containing Activated Carbon Derived From Natural Honeydew Peel Powers Aqueous Supercapacitors. Chem. Asian J..

[21] Xie Y., Xu S., Xu Z., Wu H., Deng C., Wang X. (2016). Interface-mediated extremely low thermal conductivity of graphene aerogel. Carbon.

[22] Cheng Y., Zhou S., Hu P., Zhao G., Li Y., Zhang X., Han W. (2017). Enhanced mechanical, thermal, and electric properties of graphene aerogels via supercritical ethanol drying and high-temperature thermal reduction. Sci. Rep..

[23] Hu H., Zhao Z., Wan W., Gogotsi Y., Qiu J. (2013). Ultralight and Highly Compressible Graphene Aerogels. Adv. Mater..

[24] Li C., Qiu L., Zhang B., Li D., Liu C.Y. (2016). Robust Vacuum-Air-Dried Graphene Aerogels and Fast Recoverable Shape-Memory Hybrid Foams. Adv. Mater..

[25] Peng M., Wen Z., Xie L., Cheng J., Jia Z., Shi D., Zeng H., Zhao B., Liang Z., Li T. (2019). 3D Printing of Ultralight Biomimetic Hierarchical Graphene Materials with Exceptional Stiffness and Resilience. Adv. Mater..

[26] Patil S.P., Shendye P., Markert B. (2020). Molecular Investigation of Mechanical Properties and Fracture Behavior of Graphene Aerogel. J. Phys. Chem. B.

[27] Qiu L., He Z., Li D. (2017). Multifunctional Cellular Materials Based on 2D Nanomaterials: Prospects and Challenges. Adv. Mater..

[28] Li C., Ding M., Zhang B., Qiao X., Liu C.Y. (2018). Graphene aerogels that withstand extreme compressive stress and strain. Nanoscale.

[29] Baimova J.A., Shcherbinin S.A. (2023). Strength and Deformation Behavior of Graphene Aerogel of Different Morphologies. Materials.

[30] Trembecka-Wójciga K., Sobczak J.J., Sobczak N. (2023). A comprehensive review of graphene-based aerogels for biomedical applications. The impact of synthesis parameters onto material microstructure and porosity. Arch. Civ. Mech. Eng..

[31] Ni N., Barg S., Garcia-Tunon E., Macul Perez F., Miranda M., Lu C., Mattevi C., Saiz E. (2015). Understanding Mechanical Response of Elastomeric Graphene Networks. Sci. Rep..

[32] Si Y., Wang X., Yan C., Yang L., Yu J., Ding B. (2016). Ultralight Biomass-Derived Carbonaceous Nanofibrous Aerogels with Superelasticity and High Pressure-Sensitivity. Adv. Mater..

[33] Qin Z., Jung G.S., Kang M.J., Buehler M.J. (2017). The mechanics and design of a lightweight three-dimensional graphene assembly. Sci. Adv..

[34] Lei J., Liu Z. (2018). The structural and mechanical properties of graphene aerogels based on Schwarz-surface-like graphene models. Carbon.

[35] Tang D., Ren C., Zhang L., Tao Y., Zhang P., Lv W., Jia X., Jiang X., Zhou G., Ohmura T. (2019). Size Effects on the Mechanical Properties of Nanoporous Graphene Networks. Adv. Funct. Mater..

[36] Patil S.P., Rege A., Sagardas, Itskov M., Markert B. (2017). Mechanics of Nanostructured Porous Silica Aerogel Resulting from Molecular Dynamics Simulations. J. Phys. Chem. B.

[37] Polyakova P.V., Baimova J.A. (2023). Mechanical Properties of Graphene Networks under Compression: A Molecular Dynamics Simulation. Int. J. Mol. Sci..

[38] Magnin Y., Rondepierre F., Cui W., Dunstan D., San-Miguel A. (2021). Collapse phase diagram of carbon nanotubes with arbitrary number of walls. Collapse modes and macroscopic analog. Carbon.

[39] Silveira J.F., Muniz A.R. (2018). Diamond nanothread-based 2D and 3D materials: Diamond nanomeshes and nanofoams. Carbon.

[40] Morris B., Becton M., Wang X. (2018). Mechanical abnormality in graphene-based lamellar superstructures. Carbon.

[41] Zhang B., Zhang J., Sang X., Liu C., Luo T., Peng L., Han B., Tan X., Ma X., Wang D. (2016). Cellular graphene aerogel combines ultralow weight and high mechanical strength: A highly efficient reactor for catalytic hydrogenation. Sci. Rep..

[42] Qiu L., Liu J.Z., Chang S.L., Wu Y., Li D. (2012). Biomimetic superelastic graphene-based cellular monoliths. Nat. Commun..

[43] Riaz M.A., Hadi P., Abidi I.H., Tyagi A., Ou X., Luo Z. (2017). Recyclable 3D graphene aerogel with bimodal pore structure for ultrafast and selective oil sorption from water. RSC Adv..

[44] Minakshi M., Mujeeb A., Whale J., Evans R., Aughterson R., Shinde P.A., Ariga K., Shrestha L.K. (2024). Synthesis of Porous Carbon Honeycomb Structures Derived from Hemp for Hybrid Supercapacitors with Improved Electrochemistry. ChemPlusChem.

[45] Stuart S.J., Tutein A.B., Harrison J.A. (2000). A reactive potential for hydrocarbons with intermolecular interactions. J. Chem. Phys..

[46] Orekhov N., Ostroumova G., Stegailov V. (2020). High temperature pure carbon nanoparticle formation: Validation of AIREBO and ReaxFF reactive molecular dynamics. Carbon.

[47] Zhang X., Xia W., Wang Y., Wang L., Liu X. (2024). Investigation of Projectile Impact Behaviors of Graphene Aerogel Using Molecular Dynamics Simulations. Comput. Model. Eng. Sci..

[48] LAMMPS Large-Scale Atomic/Molecular Massively Parallel Simulator. https://www.lammps.org/.

[49] Plimpton S. (1995). Fast Parallel Algorithms for Short-Range Molecular Dynamics. J. Comput. Phys..

[50] Thompson A.P., Aktulga H.M., Berger R., Bolintineanu D.S., Brown W.M., Crozier P.S., in ’t Veld P.J., Kohlmeyer A., Moore S.G., Nguyen T.D. (2022). LAMMPS—A flexible simulation tool for particle-based materials modeling at the atomic, meso, and continuum scales. Comput. Phys. Commun..

[51] VMD Molecular Graphics Viewer. https://www.ks.uiuc.edu/Research/vmd/.

[52] OVITO Open Visualization Tool. https://www.ovito.org/.

